# An In Situ Chemotherapy Drug Combined with Immune Checkpoint Inhibitor for Chemoimmunotherapy

**DOI:** 10.3390/nano13243144

**Published:** 2023-12-15

**Authors:** Xinyuan Yuan, Xiupeng Wang

**Affiliations:** 1 School of Materials Science and Engineering and Key Laboratory of Biomedical Materials of Ministry of Education, South China University of Technology, and National Engineering Research Center for Tissue Restoration and Reconstruction, Guangzhou 510641, China; 2 Health and Medical Research Institute, National Institute of Advanced Industrial Science and Technology (AIST), Central 6, 1-1-1 Higashi, Tsukuba 305-8566, Japan

**Keywords:** copper-dopped mesoporous silica (MS-Cu), disulfiram (DSF), chemoimmunotherapy, tumor environment regulation, nontoxic to toxic

## Abstract

Clinically, cancer chemotherapy still faces unsatisfactory efficacy due to drug resistance and severe side effects, including tiredness, hair loss, feeling sick, etc. The clinical benefits of checkpoint inhibitors have revived hope for cancer immunotherapy, but the objective response rate of immune checkpoint inhibitors remains around 10–40%. Herein, two types of copper-doped mesoporous silica nanoparticles (MS-Cu–1 with a diameter of about 30 nm and MS-Cu–2 with a diameter of about 200 nm) were synthesized using a one-pot method. Both MS-Cu–1 and MS-Cu–2 nanoparticles showed excellent tumor microenvironment regulation properties with elevated extracellular and intracellular ROS generation, extracellular and intracellular oxygenation, and intracellular GSH depletion. In particular, MS-Cu–2 nanoparticles demonstrated a better microenvironment modulation effect than MS-Cu–1 nanoparticles. The DSF/MS-Cu composites with disulfiram (DSF) and copper co-delivery characteristics were prepared by a straightforward method using chloroform as the solvent. Cell survival rate and live/dead staining results showed that DSF and MS-Cu alone were not toxic to LLC cells, while a low dose of DSF/MS-Cu (1–10 μg/mL) showed a strong cell-killing effect. In addition, MS-Cu–2 nanoparticles released more Cu^2+^ in a weakly acidic environment (pH = 5) than in a physiological environment (pH = 7.4), and the Cu^2+^ released was 41.72 ± 0.96 mg/L in 1 h under weakly acidic conditions. UV–visible absorption spectrometry confirmed the production of tumor-killing drugs (CuETs). The intratumoral injection of DSF/MS-Cu significantly inhibited tumor growth in vivo by converting nontoxic DSF/MS-Cu into toxic CuETs. The combination of DSF/MS-Cu and anti-CTLA–4 antibody further inhibited tumor growth, showing the synergistic effect of DSF/MS-Cu and immune checkpoint inhibitors.

## 1. Introduction

Cancer is still the leading disease that threatens the health and lives of people. In 2023, there are expected to be about 600 thousand deaths from cancer and about 2 million new cancer cases in the United States [1]. In clinical practice, surgery, chemotherapy, radiotherapy, and immunotherapy are four common approaches to treating cancer. Among them, chemotherapy is widely adopted in treating various cancers, but has deficiencies like low therapy efficacy and off-target toxicity [2,3]. Typical side effects of chemotherapy include tiredness, hair loss, feeling sick, headaches, muscle pain, stomach pain, mouth sores, blood disorders, and so on. Currently, it is still challenging to develop new antitumor drugs due to the long time, high cost, high risk, and low approval rate, while drug reuse is a faster and less expensive strategy because of its available formulation and demonstrated safety in clinic [4].

Disulfiram (DSF), which has been used to treat alcohol dependence for several decades, has showed superior antitumor effects in a variety of malignant cancers in a copper-dependent manner [5,6]. The S-S bond that exists in DSF could chelate copper to generate bis(diethyldithiocarbamate)-copper complexes (CuETs), and this exhibits a broad-spectrum antitumor effect [7,8,9,10]. Skrott’s research revealed that CuETs could induce cell death by interrupting the p97-NPL4-UFD1 pathway [5]. However, copper in the tumor microenvironment that could chelate DSF is usually inadequate, and supplementary copper is also prone to accumulation in normal tissue, leading to unwanted side effects and toxicity [7,11]. The oral administration of both DSF and copper still faces a low in vivo generation of CuET due to the poor stability and rapid metabolism of DSF, while direct CuET administration has a low water solubility problem [6]. Bagherpoor et al. [12] reported the combination of sulfasalazine and DSF-Cu inhibited lung adenocarcinoma growth in cell line and mouse models. Terashima et al. [13] reported that DSF alone inhibited Lewis lung carcinoma (LLC) progression. Wu et al. [7] designed DSF-loaded, Cu-doped hollow mesoporous silica nanoparticles (DSF@PEG/Cu-HMSNs) and found that they had high chemotherapeutic efficacy on 4T1 cells. Chen et al. [14] synthesized metal–organic frameworks (MOFs) as Cu carrier to construct DSF@HKUST drugs, which showed a tumor-responsive nontoxic-to-toxic therapeutic performance in 4T1 cells. However, complicated preparation processes were involved in these studies, which would cause synthesis difficulties and costs issues for large-scale manufacturing. In our previous study, mesoporous silica doped with copper (MS-Cu) nanospheres were confirmed to have good biocompatibility and exert good anti-tumor effects when combined with a continuous oral administration of DSF [9].

Tumor microenvironment (TME) is usually characterized by mild acidity, hypoxia, and powerful antioxidative systems [15,16], which would strongly restrict the chemotherapy and immunotherapy efficacies [17,18,19,20]. The regulatory functions of MS-Cu nanoparticles in the tumor microenvironment remain unclear. Cancer immunotherapy is used to mobilize the host’s immune system to fight cancer, enhancing the therapy efficacy in many cancer treatments [21,22,23,24,25,26]. Checkpoint blockade therapy is one of the most promising immunotherapy strategies. The common immune checkpoint inhibitors approved by the FDA include anti-cytotoxic T lymphocyte-associated protein 4 (anti-CTLA–4), anti-programmed cell death protein 1 (anti-PD–1), and anti-programmed death ligand 1 (anti-PD-L1) [27,28]. A lot of works in the literature have proved that the combination of chemotherapy and immunotherapy can exert a powerful therapeutic effect [29].

In this work, MS-Cu nanoparticles (MS-Cu–1 and MS-Cu–2) were prepared by a one-pot method according to a previous study [9]. Subsequently, a facile method using chloroform as the solvent was used to synthesize the DSF/MS-Cu composites. Finally, the TME regulatory functions (cell-killing effects, ROS generation, oxygenation, and GSH depletion) *in vitro* and the chemoimmunotherapeutic effects in vivo were investigated.

## 2. Materials and Methods

### 2.1. Preparation of MS-Cu Nanoparticles

First, 0.6 g triethanolamine (TEA, Sigma-Aldrich, St. Louis, MO, USA) was added to 66 g of 10 wt.% cetyltrimethylammonium chloride (CTAC, Tokyo Chemical Industrial Corporation Limited, Toshima, Japan) under stirring at 75 °C for 30 min. Then, 6 mL of tetraethyl orthosilicate (TEOS, FUJIFILM Wako Pure Chemical Corporation, Minato City, Japan) was dropped into the above solution within 2 min, and 0.66 g of copper nitrate (FUJIFILM Wako Pure Chemical Corporation) was added within 1 min under stirring. After 4.5 h, the precipitates were washed three times with ultrapure water and once with ethanol before being dried at room temperature for 24 h and at 75 °C for 2 h. The obtained powder was calcinated at 570 °C for 5 h and labelled MS-Cu–1.

Subsequently, 0.6 g TEA was added to 61.2 g of 2 wt.% cetyltrimethylammonium p-toluenesulfonate (CTAT, Sigma-Aldrich, St. Louis, MO, USA) under stirring at 75 °C for 30 min. Then, 4.5 mL of TEOS was dropped into the above solution within 2 min, and 0.48 g of copper nitrate was added within 1 min under stirring. After 4.5 h, the precipitates were washed three times with ultrapure water and once with ethanol before being dried at room temperature for 24 h and at 75 °C for 2 h. The resultant powder was calcinated at 570 °C for 5 h and labelled MS-Cu–2.

### 2.2. Preparation of DSF/MS-Cu Composites

First, 200 mg of DSF was dissolved in 40 mL of chloroform (FUJIFILM Wako Pure Chemical Corporation), and 400 mg MS-Cu nanoparticles were dispersed into the above solution under magnetic stirring at 25 °C for 12 h. After chloroform evaporation, the product was collected by centrifugation, followed by being washed three times with ethanol and dried at 50 °C for future use. The synthesis of DSF-Cu-containing materials usually requires complex multi-steps. In this study, a simple chloroform method was used to synthesize DSF/MS-Cu composite materials, which had the advantages of high efficiency, time saving, and easy control.

### 2.3. Characterization of MS-Cu Nanoparticles

The morphology of MS-Cu nanoparticles was observed by a transmission electron microscope (TEM, JEOL, Akishima, Japan). The Cu/Si mole ratios of the MS-Cu nanoparticles were determined by an inductively coupled plasma atomic emission spectrometer (ICP-AES, SPS7800, Seiko Instruments, Chiba, Japan). A surface area and porosity analyzer (Micromeritics Tristar II 3020, Norcross, GA, USA) was employed to characterize the surface areas of MS-Cu nanoparticles. The Cu and Si ions released from MS-Cu–2 nanoparticles at predetermined time points (1 h, 4 h, 8 h, 12 h, 24 h, 1 d, 2 d, 3 d, 4 d, 5 d) in PBS (pH = 7.4) and acetate buffer (pH = 5.0) were tested by ICP–AES. The CuET synthesized from the DSF/MS-Cu–2 composite was detected by an ultraviolet–visible (UV-vis) absorption spectrometer (V-730, JASCO, Tokyo, Japan). Zeta potentials of nanoparticles in PBS(−) were analyzed using a Delta Nano C Particle Analyzer (Beckman Coulter, Brea, CA, USA).

### 2.4. In Vitro Cytotoxicity

Briefly, 5 × 10^4^ cells/mL of Lewis lung carcinoma (LLC, Riken Bio Resource Center, Kyoto, Japan) cells were seeded onto a 96–well plate. When the cell confluence reached 80%, different concentrations (1, 2, 3, 5, and 10 μg/mL) of materials were added into each well, followed by culturing for 2 days before the cell counting kit–8 (CCK8, Dojindo Molecular Technologies, Rockville, MD, USA) was utilized to assess the cell numbers. Six replications were performed for every group to obtain the mean value and standard deviation. In addition, the cell-killing induced by materials at a concentration of 10 μg/mL was investigated by a calcein-AM (green fluorescence)/PI (red fluorescence) double staining kit according to the manufacturer’s instructions. The cell viability was observed using an inverted fluorescence microscope (IX73, Olympus, Tokyo, Japan).

### 2.5. Intracellular ROS Generation Induced by MS-Cu Nanoparticles

LLC cells (2.5 × 10^5^ cells/mL) were seeded onto a 96–well plate. When the cell confluence reached 80%, PBS was used to wash once before different concentrations (10, 20, and 50 μg/mL) of MS-Cu nanoparticles were added into each well to coculture for 6 h. The intracellular ROS generation was tested by a DCFDA/H2DCFDA-cellular ROS assay kit (Abcam, Cambridge, UK). A microplate reader (MTP–900, Corona Electric, Ibaraki, Japan) was applied to detect the fluorescence of DCF (Ex/Em = 492 nm/530 nm). The fluorescence images were captured by the inverted fluorescence microscope.

### 2.6. Extracellular ROS Generation Induced by MS-Cu Nanoparticles

Different concentrations (10, 20, and 50 μg/mL) of MS-Cu nanoparticles were dispersed into a 25 mM NaHCO_3_ buffer solution with methylene blue (MB, 10 μg/mL) and H_2_O_2_ (10 mM). After the solutions were incubated at 37 °C for determined time points (0.5, 1, and 1.5 h), an ultraviolet–visible (UV-vis) absorption spectrometer was adopted to determine the absorbance of MB at 664 nm.

### 2.7. Intracellular Oxygenation Induced by MS-Cu Nanoparticles

LLC cells (4 × 10^5^ cells/mL) were seeded onto a 96–well plate. When the cell confluence reached 80%, PBS was used to wash once, and 3 μM RDPP (Santa Cruz Biotechnology, Dallas, TX, USA) was added to coculture for 4 h. Subsequently, different concentrations (10, 20, and 50 μg/mL) of MS-Cu nanoparticles were added and the fluorescence (Ex/Em = 450 nm/630 nm) of RDPP at 1 and 3 h were detected using a microplate reader. The fluorescence images were captured by the inverted fluorescence microscope.

### 2.8. Extracellular Oxygenation Induced by MS-Cu Nanoparticles

Different concentrations (10, 20, and 50 μg/mL) of MS-Cu nanoparticles were dispersed into 3 μM RDPP, and 50 mM H_2_O_2_ was added. The fluorescence (Ex/Em = 450 nm/630 nm) of RDPP at different time points (0, 5, 10, 30, 35, 40, 45, and 50 min) was determined by applying the microplate reader.

### 2.9. Intracellular GSH Depletion of MS-Cu Nanoparticles

LLC cells (5 × 10^5^ cells/well) were seeded onto a 6-well plate. When the cell confluence reached 80%, PBS was used to wash once, and different concentrations (10, 20, and 50 μg/mL) of MS-Cu nanoparticles were added to coculture for 6 h. Then, the relative GSH/GSSH content was analyzed using a GSSG/GSH quantification kit (Dojindo Molecular Technologies, Kumamoto, Japan) following the instructions of the manufacturer.

### 2.10. In Vivo Synergistic Antitumor Effects of DSF/MS-Cu–2 Combined with Immune Checkpoint Inhibitor

The animal experiment was approved by the Ethics Committee of the National Institute of Advanced Industrial Science and Technology (AIST), Japan. Twenty female LLC tumor-bearing mice (C57BL/6JJcl, 6 weeks old, CLEA, Shizuoka, Japan) were randomly divided into 4 groups (n = 5): (i) saline (i.t.); (ii) anti–CTLA–4 (0.1 mg/mouse, i.p.); (iii) DSF/MS-Cu–2 (0.2 mg/mouse, i.t.); and (iv) DSF/MS-Cu–2 (0.2 mg/mouse, i.t.) and anti–CTLA–4 (0.1 mg/mouse, i.p.). The tumor size, tumor weight, and body weight were recorded. The tumor volume was calculated as follows: volume = 1/2 × Length × Width^2^.

### 2.11. Statistical Analysis

Student’s *t*-test was used to analyze the statistical significance between the two groups. All data were presented as mean value ± standard deviation; a value of *p* < 0.05 was considered a significant difference between the blank group and the marked group or the marked groups. * *p* < 0.5, ** *p* < 0.01, and *** *p* < 0.001.

## 3. Results and Discussion

### 3.1. Physicochemical Characterization

**Figure 1:**
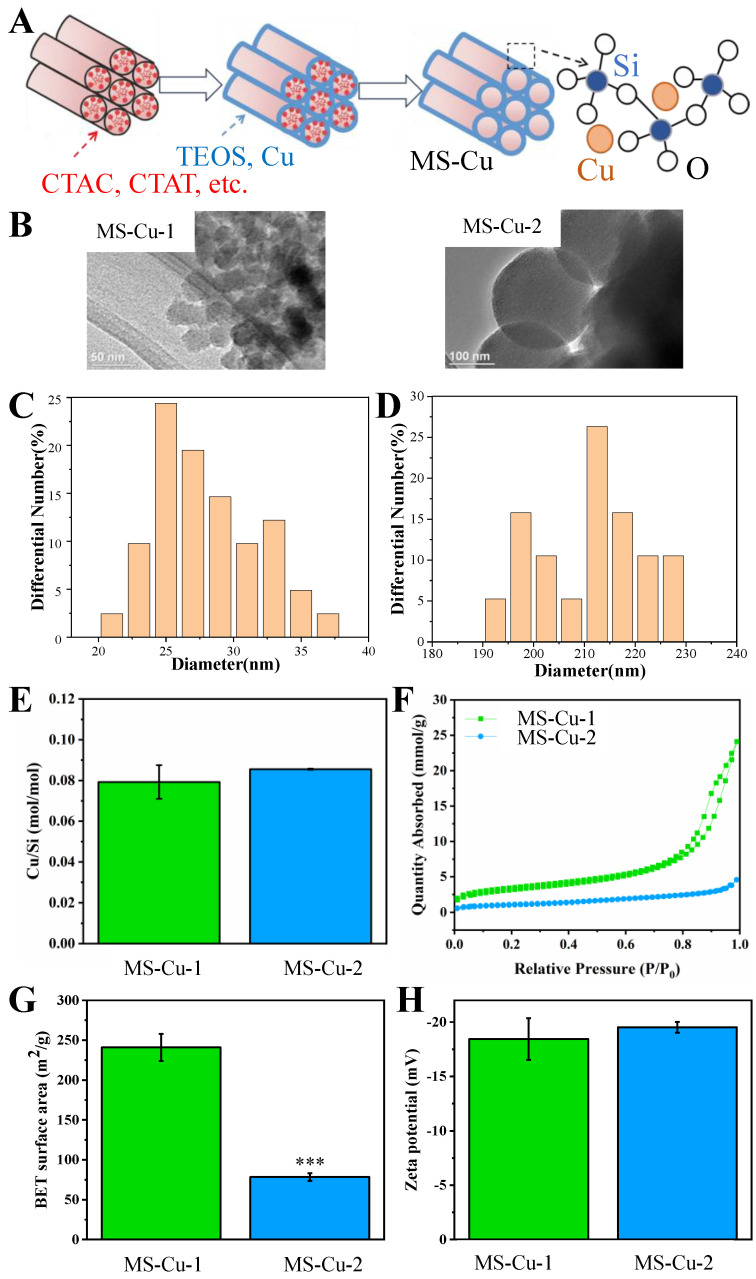
Scheme of MS-Cu synthesis (**A**), TEM images (**B**), particle size distribution of MSCu–1 (**C**) and MS-Cu–2 (**D**), Cu/Si mol ratios (**E**), N_2_ adsorption–desorption isotherms (**F**), BET surface areas (**G**), and zeta potential (**H**) of MS-Cu–1 and MS-Cu–2 nanoparticles (*** *p* < 0.001).

The TEM images (Figure 1A–D) demonstrate that the particle sizes of MS-Cu–1 and MS-Cu–2 nanoparticles were about 30 and 200 nm in diameter, respectively. Moreover, MS-Cu–2 nanoparticles were more uniform and had more regular shapes than MS-Cu-1 nanoparticles. The particle size of MS-Cu–1 and MS-Cu–2 nanoparticles can be adjusted by adjusting the TEOS concentration and the type and amount of surfactant for nanoparticle synthesis. It was reported that as the amount of TEOS increased, the particle size of MS first increased and then decreased [30,31]. The Cu/Si molar ratios of MS-Cu nanoparticles were detected via ICP. The Cu/Si molar ratios of MS-Cu–1 and MS-Cu–2 nanoparticles were 0.079 ± 0.008 and 0.086 ± 0.003, respectively (Figure 1E). As shown in Figure 1F,G, the N_2_ adsorption and desorption measurements were applied to characterize the mesoporous structure of MS-Cu nanoparticles. MS-Cu–1 and MS-Cu–2 nanoparticles showed zeta potentials of −18.44 and −19.52 mV, respectively (Figure 1H). The Brunauer–Emmett–Teller (BET) specific surface areas of MS-Cu–1 and MS-Cu–2 were 240.98 ± 16.95 and 78.37 ± 4.98 m^2^/g, respectively. The Barrett, Joyner and Halenda (BJH) adsorption cumulative surface area of pores and the BJH desorption cumulative surface area of pores for MS-Cu–1 were 217.38 and 256.24 m^2^/g, respectively. The BJH (Barrett, Joyner and Halenda) adsorption cumulative surface area of pores and BJH desorption cumulative surface area of pores for MS-Cu–2 were 74.65 and 75.04 m^2^/g, respectively. The hysteresis between adsorption and desorption isotherms is thought to be caused by a gradual desorption mechanism (known as percolation theory), owing to the combination of pores of different sizes [32].

The characterization and safety of the MS-Cu nanomaterials, although slightly different from the nanomaterials in this work, had been reported in our previous work [9]. For example, STEM-EDX images showed that Cu was uniformly immobilized in MS-Cu nanoparticles. X-ray diffraction (XRD) patterns showed the MS-Cu nanoparticles were mainly composed of amorphous silica [9]. For the side effects and safety of MS-Cu nanoparticles, (1) MS-Cu nanospheres did not show obvious cytotoxic efficacy against fibroblastic NIH3T3 cells at concentrations of 1–10 μg/mL, and (2) the MS-Cu nanospheres showed no obvious damage to the heart, kidney, liver, lung, and spleen of mice, suggesting it is unlikely to cause serious side effects in normal tissues [9].

### 3.2. In Vitro Cytotoxicity

**Figure 2:**
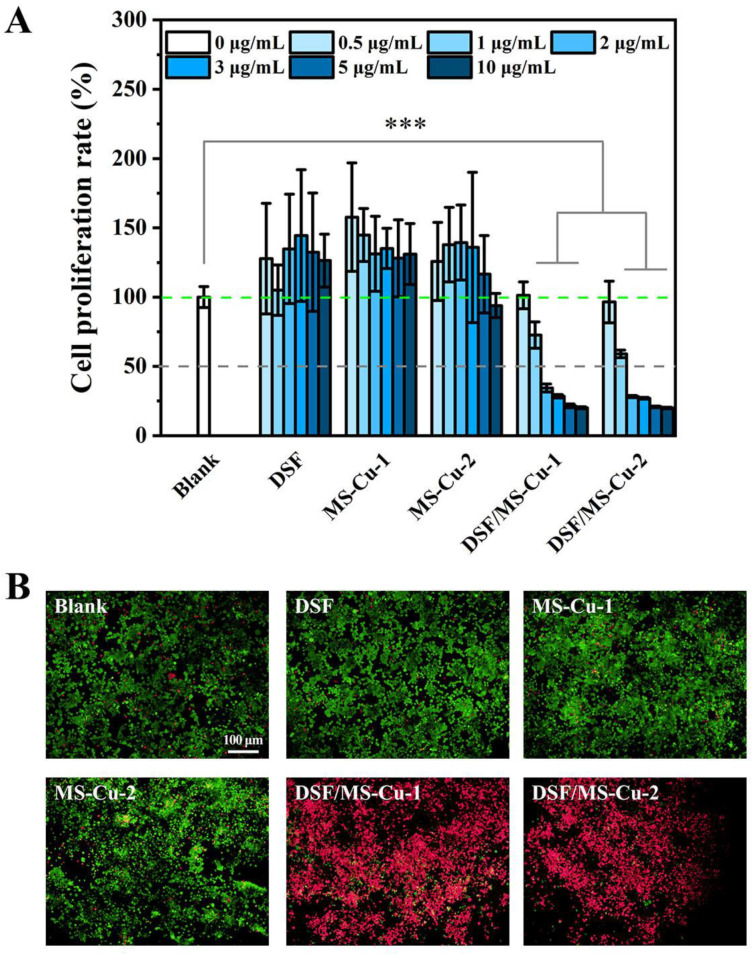
Cytotoxicity (**A**) and live/dead staining (**B**) of DSF/MS-Cu–1 and DSF/MS-Cu–2 (*** *p* < 0.001).

The CCK8 kit and live/dead staining kit were used to assess the cytotoxicity of materials. Cell proliferation results (Figure 2A) showed that DSF and MS-Cu nanoparticles at different concentrations (0.5, 1, 2, 3, 5, 10 μg/mL) had no obvious cell-killing effects. Nevertheless, DSF/MS-Cu composites demonstrated a high toxicity to LLC cells even at a low concentration (1 μg/mL). A similar phenomenon was also observed in live and dead staining images (Figure 2B). DSF/MS-Cu composites at 10 μg/mL displayed a strong red fluorescence signal, while the other four groups only displayed a weak red fluorescence signal. Wu et al. [7] constructed a codelivery system (DSF@PEG/Cu-HMSNS) that possessed superior 4T1 cell-killing efficacy. Wang [9] reported that the direct combination of MS-Cu nanospheres and DSF manifested obvious cytotoxicity to mouse oral squamous cell carcinoma 1 (MOC1) and MOC2. The results in this study suggested that the composites of DSF and MS-Cu also had excellent potency for LLC cell killing.

### 3.3. ROS Generation

**Figure 3:**
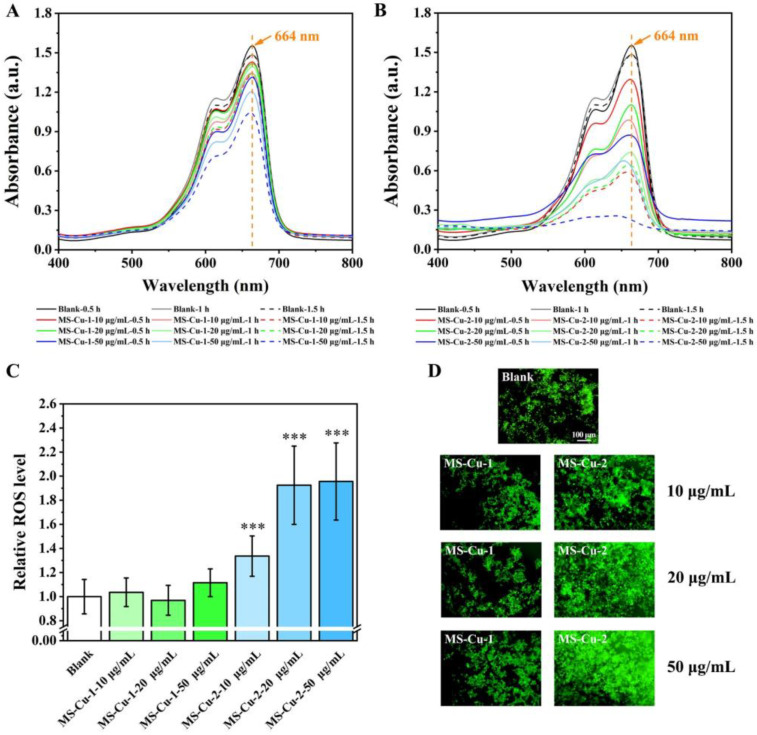
Extracellular ROS generation (**A**,**B**), intracellular ROS generation (**C**), and fluorescence images of intracellular ROS level in LLC cells (**D**) induced by MS-Cu–1 and MS-Cu–-2 nanoparticles (*** *p* < 0.001).

The generation of ROS via a Fenton-like reaction during the chelation of DSF and Cu^2+^ was found to be one of the reasons for tumor cell apoptosis [7,33]. MS-Cu nanospheres were found to upregulate the ROS level [7]. The chemodynamic activity (Fenton-like reaction) could induce ∙OH generation, which could be reflected by the degradation of MB [34]. The extracellular ROS (∙OH) produced by MS-Cu nanoparticles was detected by the concentration of MB over time in a NaHCO_3_ buffer solution containing H_2_O_2_. The intracellular ROS level of LLC cells induced by MS-Cu nanoparticles was determined by a DCFDA/H2DCFDA-cellular ROS assay kit. The extracellular and intracellular ROS determinations (Figure 3) showed that MS-Cu–2 nanoparticles at 10, 20, and 50 μg/mL could generate ROS effectively, while the ROS generation of MS-Cu–1 nanoparticles at 10, 20, and 50 μg/mL was not obvious compared to that of MS-Cu–2 nanoparticles. Specifically, the peak of MB at 664 nm decreased in a time- and concentration-dependent manner in the extracellular ROS results (Figure 3A,B). At 0.5 h, the absorbances of MB in 10, 20, and 50 μg/mL of MS-Cu–1 nanoparticles were 92%, 91%, and 85% of that of the blank, respectively, while the absorbances of MB in 10, 20, and 50 μg/mL of MS-Cu–2 nanoparticles were 83%, 71%, and 56% of that of the blank, respectively. When the incubation time was prolonged to 1.5 h, the absorbance of MB in 10, 20, and 50 μg/mL MS-Cu–1 nanoparticles further decreased to 89%, 91%, and 70% of that of the blank, respectively, while the absorbance of MB in 10, 20, and 50 μg/mL MS-Cu–2 nanoparticles further decreased to 39%, 43%, and 15% of that of the blank, respectively. From the intracellular results (Figure 3D), MS-Cu–2 nanoparticles at 10, 20, and 50 μg/mL emitted stronger ROS fluorescence, and the relative intracellular ROS levels were also upregulated significantly compared to those of the blank (Figure 3B). These intracellular ROS results (Figure 3C,D) were consistent with the extracellular ROS results (Figure 3A,B), indicating that MS-Cu–2 nanoparticles had a higher ROS generation capacity than MS-Cu–1 nanoparticles. MS-Cu–2 nanoparticles generated ROS (∙OH) by a Fenton-like reaction, and higher concentrations of MS-Cu–2 nanoparticles showed stronger ROS (∙OH) generation.

### 3.4. Oxygenation

**Figure 4:**
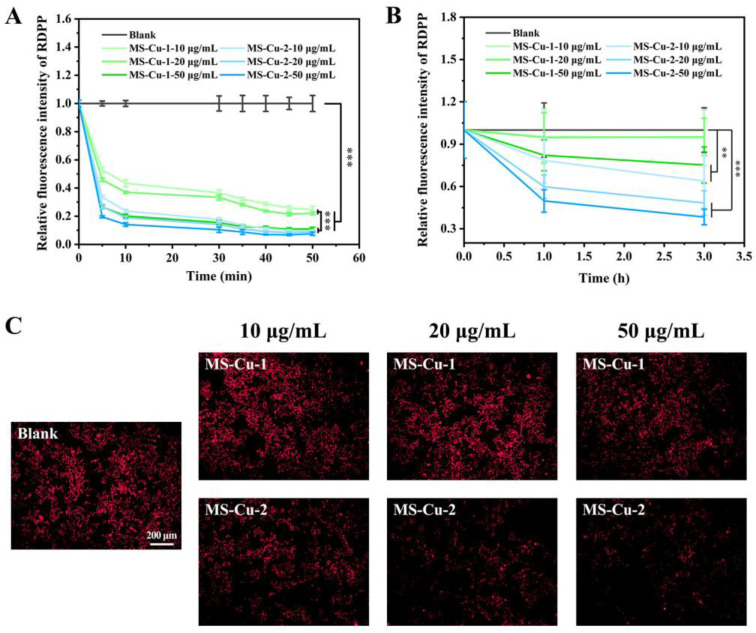
Extracellular oxygenation (**A**) and intracellular oxygenation (**B**) ability, and fluorescence images of intracellular oxygenation level in LLC cells stained with RDPP (**C**) (** *p* < 0.01, *** *p* < 0.001).

The oxygenation level could be reflected by the decrease in RDPP [35]. The extracellular and intracellular oxygenation in different concentrations (10, 20, and 50 μg/mL) of MS-Cu nanoparticles also demonstrated a time- and concentration-dependent manner. In extracellular oxygenation results (Figure 4A), the content of RDPP in 10, 20, and 50 μg/mL of MS-Cu–1 nanoparticles dramatically decreased to 53%, 46%, and 27% compared with that of blank, while the content of RDPP in 10, 20, and 50 μg/mL of MS-Cu–2 nanoparticles dramatically decreased to 34%, 27%, and 20% compared with that of the blank in 5 min. Until the end time point (50 min), the content of RDPP in 10, 20, and 50 μg/mL of MS-Cu–1 nanoparticles decreased to 25%, 22%, and 11% compared with that of the blank, while the content of RDPP in the MS-Cu–2 group decreased to below 10% compared with that of the blank. As for the intracellular oxygenation levels (Figure 4B), the RDPP intensities in the MS-Cu–2 group clearly decreased in comparison with those of the blank, while the RDPP intensity apparently decreased only at 50 μg/mL in the MS-Cu–1 group. The RDPP fluorescence images (Figure 4C) showed a similar trend to the intracellular oxygenation levels (Figure 4B). All these results indicated that MS-Cu nanoparticles had excellent oxygenation ability and MS-Cu–2 showed a higher oxygenation ability than MS-Cu–1. Hypoxia in the tumor microenvironment was one of the reasons to restrict the chemoimmunotherapeutic effects [36,37]. Therefore, the excellent oxygenation ability of MS-Cu nanoparticles could reverse the hypoxia, thus helping to enhance the effects of chemoimmunotherapy.

### 3.5. Intracellular GSH Depletion

**Figure 5:**
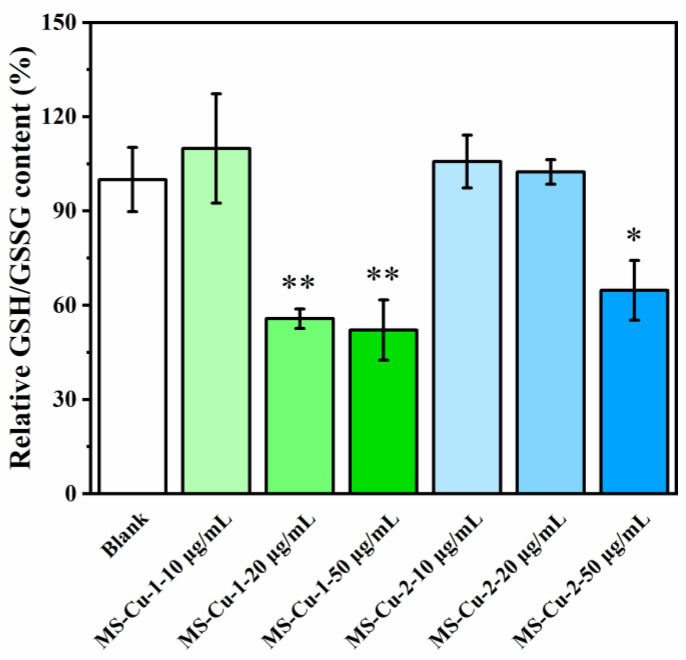
Intracellular relative GSH/GSSG content in LLC cells incubated with MS-Cu–1 and MS-Cu–2 nanoparticles (* *p* < 0.5, ** *p* < 0.01).

The intracellular GSH’s depletion ability was further investigated by co-culturing LLC cells with MS-Cu nanoparticles for 6 h, and then the GSSH/GSH quantification kit was employed to measure the relative GSH/GSSH content. The results (Figure 5) showed that MS-Cu–1 at concentrations of 20 and 50 μg/mL and MS-Cu–2 at a concentration of 50 μg/mL significantly reduced the relative GSH/GSSH content, indicating that MS-Cu nanoparticles could convert GSH (a reduced state) into GSSH (an oxidized state) in LLC cells. Glutathione (GSH) is an antioxidant that plays an important role in maintaining redox status and protecting against oxidative stress. Works in the literature showed that GSH depletion could decrease ROS consumption and increase H_2_O_2_ in the tumor microenvironment, which would be an indirect enhancement to ROS-based antitumor therapies [38,39]. Previous studies have also shown that GSH depletion showed a synergistic effect with chemodynamic therapy, photothermal therapy, and photodynamic therapy [40].

### 3.6. Ion Release and the Generation of CuET

**Figure 6:**
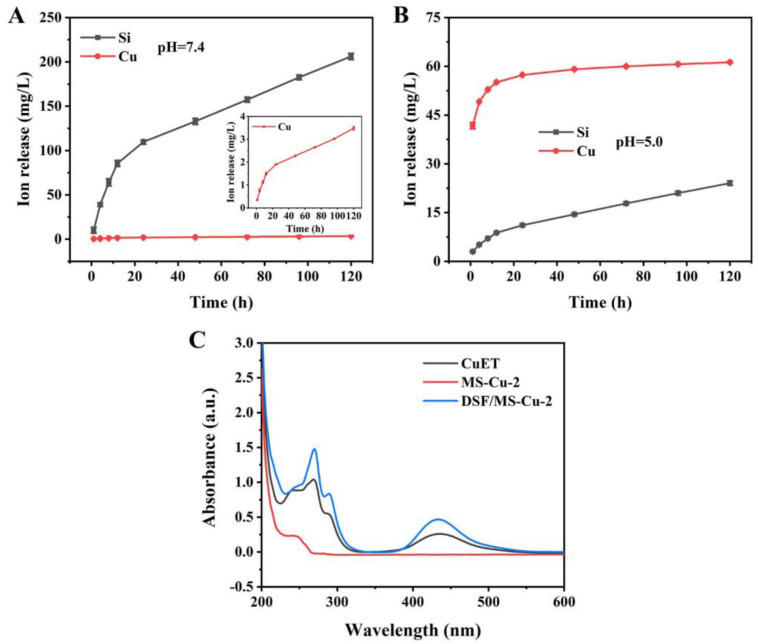
Ion release (**A**) of MS-Cu–2 nanoparticles soaked in PBS (pH = 7.4), ion release (**B**) of MS-Cu–2 nanoparticles soaked in acetate buffer (pH = 5.0), and UV-vis absorption (**C**) of CuET, MS-Cu–2, and DSF/MS-Cu–2 in ethanol.

Based on the above results, it can be seen that the composite of DSF and MS-Cu nanoparticles demonstrated strong tumor cytotoxicity and MS-Cu had outstanding tumor microenvironment regulation abilities (stronger ROS generation, enhanced oxygenation, and good GSH depletion). In addition, MS-Cu–2 nanoparticles showed a stronger tumor cell-killing efficacy and higher tumor microenvironment regulation ability than MS-Cu–1 nanoparticles. Therefore, MS-Cu–2 was selected for the following experiments. Combined with the works reviewed [8,10,11,13], it is speculated that the released DSF and Cu ion from DSF/MS-Cu generated the toxic product, CuET, bringing excellent anti-tumor activity. As a consequence, the ion release of MS-Cu–2 nanoparticles and the generation of CuET were detected by ICP and the UV–vis absorption spectrometer, respectively. The cumulative ion release results (Figure 6A,B) in PBS (pH = 7.4) and acetate buffer (pH = 5) solutions showed that MS-Cu–2 nanoparticles sustainably released Cu and Si ions during the 5 d test period. In the mildly acidic condition (pH = 5), the Cu release from MS-Cu-2 nanoparticles could reach 41.72 ± 0.96 mg/L at 1 h, while it only reached 0.353 ± 0.002 mg/L in the physiological condition at 1 h. Previous research [9] revealed that the Cu-O bond in MS-Cu could be attacked and broken by H^+^, which is a typical feature of TME. In addition, it was found that MS-Cu–2 nanoparticles released more Cu ions in a mildly acidic environment (pH = 5) than in a physiological one (pH = 7.4) throughout the whole test period, which is in favor of synthesizing the tumor-specific killer (CuET) in situ while avoiding off-target toxicity to normal tissues. The UV-vis absorption spectra (Figure 6C) displayed two characteristic absorption peaks (265 and 425 nm) of CuET, proving that the DSF molecule and Cu ion released from the DSF/MS-Cu–2 composite could form the toxic metabolite CuET successfully.

### 3.7. In Vivo Anti-Tumor Efficacy of DSF/MS-Cu–2 in Combination with the Anti-CTLA–-4 Antibody

**Figure 7:**
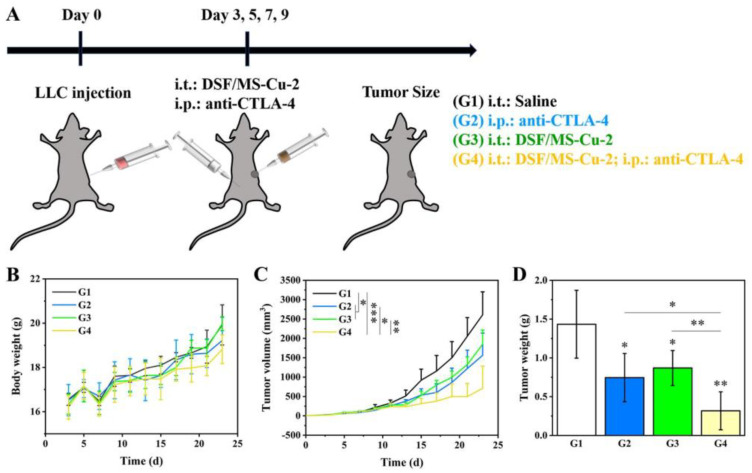
Experimental protocol (**A**), body weight (**B**), tumor volume (**C**), and tumor weight (**D**) (* *p* < 0.5, ** *p* < 0.01, and *** *p* < 0.001).

**Scheme 1:**
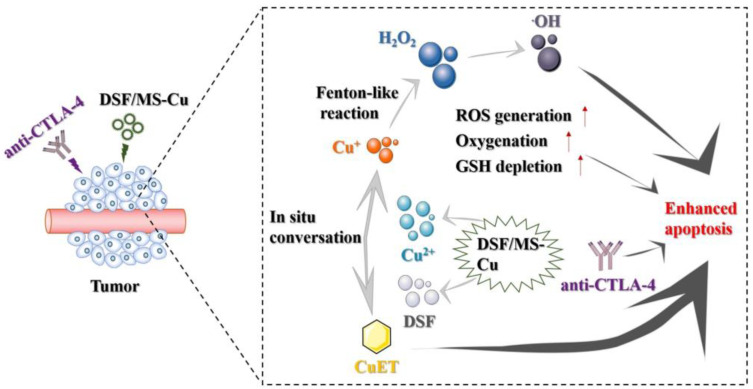
Synergistic anti-tumor efficacy of DSF/MS-Cu and immune checkpoint inhibitors.

In light of the excellent in vitro tumor cell toxicity, ROS generation, oxygenation, and GSH depletion effects of DSF/MS-Cu–2 and MS-Cu–2 nanoparticles, the in vivo anti-tumor efficacy was evaluated by DSF/MS-Cu–2 intratumor injection in combination with anti-CTLA–4 antibody intraperitoneal injection. The animal experiment protocol is shown in Figure 7A. Figure 7B showed that no obvious difference in mouse body weights was observed among the four groups, indicating that these treatments caused neglectable toxicity to mice. As shown in Figure 7C,D, DSF/MS-Cu–2 and the anti-CTLA–4 antibody treatments clearly inhibited tumor growth compared to the saline alone group, but there was no significant difference between these two groups. Moreover, the combination group dramatically suppressed the growth of the tumor compared to the saline alone group. The tumor volume and tumor weight of the combination group were 710 ± 575 mm^3^ and 0.318 ± 0.24 g, while those of the untreated group (saline) were 2610 ± 592 mm^3^ and 1.434 ± 0.44 g, respectively. Notably, the combination of DSF/MS-Cu–2 and the anti-CTLA–4 antibody significantly inhibited tumor growth compared to DSF/MS-Cu and anti-CTLA–4 antibody groups, indicating that the combination of DSF/MS-Cu–2 and the anti-CTLA–4 antibody exhibited a synergistic enhancement in inhibiting tumor growth. Taken together, DSF/MS-Cu–2 alone demonstrated antitumor efficacy, and the combination of DSF/MS-Cu–2 and the anti-CTLA–4 antibody can play a synergistic role in inhibiting tumor growth with chemoimmunotherapy (Scheme 1). Although immune checkpoint inhibitors bring new hope for cancer treatment, their effectiveness is only 10–40% [33,34]; this study showed that the combined use of DSF/MS-Cu–2 and the immune checkpoint inhibitor showed a synergistic anti-tumor efficacy.

## 4. Conclusions

In summary, MS-Cu nanoparticles were successfully synthesized through a one-pot method, and DSF and MS-Cu composite materials (DSF/MS-Cu) were constructed that can efficiently co-release Cu and DSF in situ to form tumor-killing CuETs. MS-Cu nanoparticles had excellent tumor microenvironment regulatory functions with elevated extracellular and intracellular ROS generation, extracellular and intracellular oxygenation, and intracellular GSH depletion. The cell survival rate and live/dead staining results showed that DSF and MS-Cu alone were not toxic to LLC cells, while 1 μg/mL DSF/MS-Cu showed a strong cell killing effect. In addition, MS-Cu nanoparticles released more Cu^2+^ in a weakly acidic environment (pH = 5) than in the physiological environment (pH = 7.4), and released Cu^2+^ reached 41.72 ± 0.96 mg/L in 1 h under weakly acidic conditions. UV–visible absorption spectrometry confirmed the production of tumor-killing CuETs. The intratumoral injection of DSF/MS-Cu significantly inhibited tumor growth in vivo. The combination of DSF/MS-Cu and anti-CTLA–4 antibody further inhibited tumor growth, showing the synergistic effect of DSF/MS-Cu and immune checkpoint inhibitors. Further studies on Cu metabolism in vivo and a detailed mechanism analysis of MS-Cu nanoparticles in chemoimmunotherapy are required for clinical applications.

## Data Availability

Data will be made available on request.
